# A comparative *in vivo* study of natural sweetening agents and their effect on salivary pH levels among children visiting a dental hospital in North India

**DOI:** 10.6026/973206300213917

**Published:** 2025-10-31

**Authors:** Payal Paul, Sandeep Kumar Shah, Shambhavi Dixit, Mohammed Irfaan, Bhawana Tiwari, Revathy Seetharaman, Swati Dwivedi

**Affiliations:** 1 Department of Paediatric & Preventive Dentistry, Career Post Graduate Institute Of Dental Sciences and Hospital, Lucknow, Uttar Pradesh, India; 2 Department of Orthodontics and Dentofacial Orthopaedics, Vinayaka Mission's Sankarachariyar Dental College, A Constituent College of Vinayaka Mission's Research Foundation (Deemed to be University), Ariyanoor, Tamilnadu, India

**Keywords:** Natural sweeteners, salivary pH, children, dental caries

## Abstract

Natural sweetening agents have recently gained attention as potential alternatives to refined sugar, owing to their possible non-cariogenic or even protective effects against dental caries. Therefore, it is of interest to evaluate the effect of natural sweetening agents on salivary pH levels in children visiting a dental hospital in North India. This current *in vivo* study was conducted among 140 children who were randomly allocated to four groups (n = 35 each): Group A (xylitol), Group B (maple syrup), Group C (date sugar) and Group D (distilled water, control). Saliva was collected at baseline, 0, 15 and 30 minutes post-rinsing and pH was assessed using indicator strips. All natural sweeteners significantly increased salivary pH (p < 0.05), peaking at 15 minutes. Xylitol showed the highest mean pH at 30 minutes (7.40 ± 0.60), while no significant changes occurred in the control group. Xylitol, maple syrup and date sugar positively modulate salivary pH, with xylitol showing the greatest effect.

## Background:

Globally, dental caries is a leading chronic disease affecting all age groups. According to the Global Oral Health Data Bank (WHO), the prevalence of dental caries in children and adults can range from 49% to 83%, depending on the country and population group [1]. Factors such as dietary habits (high sugar intake), socioeconomic disparities and accessibility to dental care services likely contribute to this upward trend [2]. Among these, the role of sugars has widely contributed to the deterioration of oral health among the population, irrespective of age, gender and geography. When consumed, sugars are broken down by bacteria in dental plaque, producing acids that lower the pH in the mouth. This acidic environment causes demineralization of tooth enamel and dentin, leading to cavities over time. Frequent intake of sugary foods and drinks allows bacteria to thrive, making plaque more harmful and reducing the natural protective effect of saliva. In addition, high sugar consumption indirectly worsens gum health by encouraging plaque accumulation and is linked with risk factors like obesity and diabetes, which can aggravate periodontal disease [3, 4]. Notable studies, such as the Vipeholm Study, highlighted that the frequency and consistency of sugar intake play a bigger role in cavity development than the overall amount of sugar consumed. Frequent consumption of sugary foods, especially in sticky forms, results in longer exposure to acid and a greater risk of tooth decay [5]. In recent years, sugar substitutes have gained popularity due to their lower calorie content. These alternatives provide sweetness without being broken down by cavity-causing bacteria, which means they do not contribute to acid production or lower the pH of dental plaque.

The search for sucrose alternatives has led to the creation of artificial sweeteners, such as aspartame, saccharin, cyclamate, xylitol and mannitol, many of which are considered to have low cariogenic potential. Ongoing research has also shifted focus toward finding and assessing natural sweeteners that are safer for dental health. This has contributed to a growing interest in the use of natural products in dentistry. Among the commonly used natural sweeteners like licorice, dates, jaggery, maple syrup and xylitol-a naturally derived compound often used as an artificial sweetener in dental products [6, 7-8]. Although artificial sweeteners and their dental benefits have been studied, comparative data on natural sweeteners and their immediate influence on salivary pH in children are scarce in the Indian context. Few substantial studies have specifically assessed children visiting dental hospitals in North India, a population that often presents with a high burden of dental caries. Generating such evidence will not only contribute to preventive dentistry but may also support public health recommendations for safer dietary alternatives to refined sugar. Therefore, it is of interest to evaluate of the effect of natural sweetening agents on salivary pH levels in children, thereby providing insights into their potential role in reducing caries risk and promoting better oral health practices.

## Materials and Methods:

The study was conducted in the Department of Pediatric and Preventive Dentistry at Career Post Graduate Institute of Dental Sciences and Hospital, Uttar Pradesh. Ethical approval was obtained from the Institutional Ethical Committee before the initiation of the research. The study protocol adhered to the principles outlined in the Declaration of Helsinki (revised 2013). Written informed consent was obtained from the parents or legal guardians of all participating children, who were recruited in accordance with predefined inclusion and exclusion criteria.

## Inclusion criteria:

[1] Children within the 6-14 year age range.

[2] Participants must be free from active dental caries.

[3] Only medically healthy children with no systemic illnesses will be considered.

[4] Children who have not received any form of preventive dental care in the past.

## Exclusion criteria:

[1] Children who are currently receiving any form of dental treatment.

[2] Those taking medications that could impact salivary secretion.

[3] Individuals who regularly use mouth rinses as part of their oral hygiene routine.

[4] Children with painful or disabling oral health issues.

[5] Participants with a known systemic medical condition.

[6] Children who are unable to understand or follow the study instructions.

## Group allocation:

A total of 140 children were randomly assigned to one of four groups: three experimental groups (Group A, Group B and Group C), each receiving a different mouth rinse solution and one control group (Group D).

Group A: Xylitol dissolved in distilled water (n = 35)

Group B: Maple syrup dissolved in distilled water (n = 35)

Group C: Dates Jaggery dissolved in distilled water (n = 35)

Group D: Distilled water (n = 35)

## Preparation of the solution:

Each mouth rinse solution was freshly mixed before use. The experimental solutions were made using commercial forms of xylitol, maple syrup and date sugar. For preparation, 10 grams of the respective sweetening agent was combined with 90 milliliters of distilled water based on the group assignments.

## Saliva collection procedure:

Following participant selection, children were randomly allocated to the designated groups. To ensure consistency, they were advised to abstain from eating for at least one hour before sample collection. Participants were then asked to allow saliva to pool in the mouth for one minute and subsequently expel it into a sterile collection container. The saliva samples were then evaluated for their pH levels.

## pH Assessment:

Unstimulated saliva samples, collected before rinsing, were stored in sterile collection vials. The pH level of each sample was determined using commercially available pH indicator strips (IONIX, Perfect Sales India, NIT Faridabad, Haryana - 121001). A strip was gently inserted into the saliva sample and left for approximately 10 seconds. Any color shift observed on the strip was then compared against the standard reference scale provided by the manufacturer. The corresponding pH value was noted and recorded for analysis. Following the initial measurement of baseline pH, each participant was provided with 90 mL of the freshly prepared solution specific to their group. They were instructed to rinse their mouth with the solution for one minute before swallowing it. Subsequent salivary pH readings were taken at baseline (0 minutes), then at 15 minutes and 30 minutes post-consumption.

## Statistical analysis:

The data for this study were entered and organized using Microsoft Excel 2013. Statistical analysis was conducted using SPSS version 25. Descriptive statistics, including the mean and standard deviation, were calculated. One-way analysis of variance (ANOVA) was employed to evaluate statistical differences.

## Results:

The mean salivary pH values at baseline, immediately after consumption (0 min) and at 15 and 30 minutes for the different groups are presented in Table 1 (see PDF). All three natural sweetening agents-xylitol, maple syrup and date sugar-produced a significant increase in salivary pH from baseline, with values peaking at 15 minutes and remaining above baseline at 30 minutes (p < 0.05). Among these, xylitol showed the highest mean salivary pH at 30 minutes (7.40 ± 0.60). Maple syrup and date sugar demonstrated comparable effects, with mean salivary pH values of 7.35 ± 0.49 and 7.32 ± 0.60, respectively, at 30 minutes. In contrast, the distilled water group did not show any significant change in salivary pH across the observation period (p = 0.67). These findings suggest that natural sweetening agents, particularly xylitol, can positively modulate salivary pH in children.

## Discussion:

Dental caries continues to be one of the most prevalent chronic diseases worldwide, exerting a heavy burden on health systems and affecting individuals across all ages [9, 10]. The disease process arises from a complex interplay between tooth surfaces, acidogenic bacteria and a diet rich in fermentable carbohydrates [6]. Among all oral microorganisms, Streptococcus mutans and related species play a dominant role due to their ability to form biofilms, thrive in low pH conditions and metabolize sugars into acids that demineralize enamel [7, 8 and 11]. The pioneering Vipeholm and Gustafsson studies further demonstrated that the frequency and physical form of carbohydrate intake are critical determinants of caries development rather than the total sugar consumed [5]. In the present study, children aged 6-14 years were selected because this stage encompasses mixed dentition and early permanent dentition, periods associated with high caries susceptibility. Salivary pH analysis in this age group provides valuable insights into the immediate effects of sweeteners on the oral environment. Our findings demonstrated that all the three natural sweeteners-xylitol, maple syrup and date sugar-produced a significant rise in salivary pH, peaking at 15 minutes and remaining above baseline at 30 minutes, whereas the control group (distilled water) showed no such effect. Xylitol (Group A) demonstrated the highest salivary pH at 30 minutes (7.40 ± 0.60). This aligns with an earlier study by Sahni *et al.* which confirmed the anticariogenic potential of xylitol [12]. Xylitol is not metabolized by cariogenic bacteria like *S. mutans*, thereby preventing acid production, reducing bacterial adhesion and lowering plaque accumulation [13]. Furthermore, xylitol stimulates salivary flow, enhancing calcium and phosphate deposition, which favors enamel remineralization [2, 4]. These mechanisms explain why xylitol is consistently recognized as the most effective sugar substitute in pediatric dentistry [13]. Maple syrup (Group B) also increased salivary pH (7.35 ± 0.49 at 30 minutes). While maple syrup contains sugars, its lower glycemic index compared to sucrose and the presence of trace minerals and polyphenols may contribute to its less cariogenic profile. Although not entirely safe for dental health, maple syrup may serve as a better alternative to refined sugar in moderation. Its effect can be linked with findings from sucrose substitution studies, where sugar alternatives reduced the cariogenic potential of dental biofilms [3, 8]. Nevertheless, good oral hygiene remains essential to mitigate the inherent risks of free sugar intake. Date sugar (Group C) produced a similar but slightly lower increase in salivary pH (7.32 ± 0.60 at 30 minutes). Date sugar is rich in antioxidants and polyphenols, which exert anti-inflammatory and antimicrobial effects that may benefit oral health. These findings are parallel to several honey studies, where natural sugars with phytochemical components demonstrated antibacterial activity against oral pathogens. For example, Lin *et al.* [14] and Molan [15] showed the broad-spectrum antibacterial effects of manuka honey, while Irish *et al.* [16], Cooper *et al.* [17], Patel *et al.* [18] and Sela *et al.* [19] confirmed honey's ability to inhibit *S. mutans*, improve enamel microhardness and modulate salivary microbial counts. Although date sugar differs in composition from honey, its similar antioxidant profile may underlie its positive influence on salivary pH and possible protective role in caries prevention.

## Conclusion:

Natural sugar substitutes like xylitol, maple syrup and date sugar can reduce caries risk in children is reasonable, particularly for xylitol, which has robust scientific backing. Maple syrup and date sugar may offer some advantages over refined sugar due to their natural components and potential antimicrobial effects. However, their benefits are best realized when combined with good oral hygiene practices and a balanced diet. To extrapolate the findings of this short study, there is a need for a larger sample size to determine the effect of natural sugar substitutes on salivary pH.
